# Disrupted Intraregional Brain Activity and Functional Connectivity in Unilateral Acute Tinnitus Patients With Hearing Loss

**DOI:** 10.3389/fnins.2019.01010

**Published:** 2019-09-19

**Authors:** Gang-Ping Zhou, Xin-Yi Shi, Heng-Le Wei, Li-Jie Qu, Yu-Sheng Yu, Qing-Qing Zhou, Xindao Yin, Hong Zhang, Yue-Jin Tao

**Affiliations:** ^1^Department of Radiology, The Affiliated Jiangning Hospital of Nanjing Medical University, Nanjing, China; ^2^Department of ENT, The Affiliated Jiangning Hospital of Nanjing Medical University, Nanjing, China; ^3^Department of Radiology, Nanjing First Hospital, Nanjing Medical University, Nanjing, China

**Keywords:** tinnitus, fMRI, fALFF, ReHo, FC, DMN

## Abstract

**Purpose:**

The present study combined fractional amplitude of low-frequency fluctuations (fALFF), regional homogeneity (ReHo), and functional connectivity (FC) to explore brain functional abnormalities in acute tinnitus patients (AT) with hearing loss.

**Methods:**

We recruited twenty-eight AT patients and 31 healthy controls (HCs) and ran resting-state functional magnetic resonance imaging (fMRI) scans. fALFF, ReHo, and FC were conducted and compared between AT patients and HCs. After that, we calculated correlation analyses among abnormal fALFF, ReHo, FC, and clinical data in AT patients.

**Results:**

Compared with HCs, AT showed increased fALFF values in the right inferior temporal gyrus (ITG). In contrast, significantly decreased ReHo values were observed in the cerebellar vermis, the right calcarine cortex, the right precuneus, the right supramarginal gyrus (SMG), and the right middle frontal gyrus (MFG). Based on the differences in the fALFF and ReHo maps, the latter of which we defined as region-of-interest (ROI) for FC analysis, the right ITG exhibited increased connectivity with the right precentral gyrus. In addition, the right MFG demonstrated decreased connectivity with both the bilateral anterior cingulate cortex (ACC) and the left precentral gyrus.

**Conclusion:**

By combining ReHo, fALFF, and FC analyses, our work indicated that AT with hearing loss had abnormal intraregional neural activity and disrupted connectivity in several brain regions which mainly involving the non-auditory area, and these regions are major components of default mode network (DMN), attention network, visual network, and executive control network. These findings will help us enhance the understanding of the neuroimaging mechanism in tinnitus populations. Moreover, these abnormalities remind us that we should focus on the early stages of this hearing disease.

## Introduction

Tinnitus, a common hearing disorder, is often described as a ringing, buzzing, or hissing sensation without any corresponding external auditory source (Jastreboff, 1990; Moller, 2007). It affects approximately 10–15% of the population, especially adults over the age of 65 (Eggermont and Roberts, 2004). Tinnitus can be divided into objective and subjective tinnitus. In rare cases, an objective source can be identified that is susceptible to treatment. However, in many of cases, tinnitus is subjective which is a kind of feeling that cannot be perceived by others. Moreover, tinnitus is related to hearing loss in the majority of cases, as both symptoms often occur together (Mühlnickel et al., 1998). People with tinnitus often suffer from sleep disorders, depression, and anxiety, conditions that reduce the quality of life (Reynolds et al., 2004; Baguley et al., 2013). The tinnitus symptoms can be acute or chronic. In clinical practice, we noticed that acute tinnitus patients (AT) are much more eager to seek for clinical help than chronic tinnitus patients. However, the underlying pathophysiology of tinnitus is still poorly understood.

According to previous studies, the central nervous system (CNS) is believed to play an important role in the pathophysiology of tinnitus (Lockwood et al., 2002; Eggermont, 2005; Bartels et al., 2007). Based on electrophysiological and neuroimaging studies (Lockwood et al., 1998; Kaltenbach et al., 2005), aberrant neural activity in the central auditory pathway may generate tinnitus by a range of mechanisms, such as altered neural synchrony, dysfunctional noise canceling, increased central noise, tonotopic map reorganization, upregulation of spontaneous firing rates, and aberrant neural connectivity between auditory and non-auditory structures. Specifically, the central auditory pathway appears to increase its gain to compensate for the reduced sensorineural input from the cochlea, which results in hyperactivity in the auditory pathway (Eggermont and Roberts, 2004). The neural synchrony increased after a noise-induced hearing loss, particularly for neurons representing the affected part of the tonotopic array, which tends to be spatially coincident with changes in the frequency tuning properties of the same affected neurons (Soleymani et al., 2011). A proposed model suggests that the tinnitus sensation might reach conscious awareness only when aberrant neuronal activity in the primary sensory cortex is connected to a broader cortical network involving frontal, parietal, and limbic regions (De Ridder et al., 2011a). Generally, the noise signal of tinnitus is identified by the limbic system and eliminated from perception (“tuned out”) by feeding it back to the (inhibitory) thalamic reticular nucleus, which subtracts it from the afferent auditory signal. When the limbic regions become dysfunctional, noise-cancelation breaks down and the tinnitus signal permeates to the auditory cortex (Rauschecker et al., 2010). Additionally, the thalamic dysrhythmia, a disruption in the normal relay mechanism of the thalamus to the auditory cortex, also contributes the generation of the tinnitus percept (De Ridder et al., 2011b). As subjective tinnitus is an endogenous ongoing process, the neural network has been proven changing over time (Vanneste et al., 2011). Therefore, a better characterization of altered central neural processes in acute tinnitus can offer a better understanding of its physiopathology and may contribute to the development of therapeutic intervention procedures.

Since subjective tinnitus is characterized by highly subjective, intrinsic, and persistent sound hallucinations (Schecklmann et al., 2014), resting-state functional magnetic resonance imaging (rs-fMRI) has proven to be a powerful and non-invasive technique *in vivo* which reflected in the low-frequency (0.01–0.1 Hz) fluctuations in blood-oxygenation-level dependent (BOLD) signals (Mantini et al., 2007; Husain and Schmidt, 2014). Amplitude of low-frequency fluctuation (ALFF) reflects the intensity of regional neuronal activity by measuring intrinsic brain responses at the baseline state (Biswal et al., 1995). It has been proven as an effective method in assessing the altered neural activity of various neurological or psychiatric disorders (Hoptman et al., 2010; Zhang et al., 2010; Wang et al., 2011; Chen et al., 2012a). The pulsatile tinnitus patients have been reported significantly increased ALFF in bilateral precuneus and inferior frontal gyrus and decreased ALFF in multiple occipital area (Han et al., 2014). Another fMRI study using ALFF method reported significantly increased ALFF values within several selected regions which were correlated positively with tinnitus distress and duration (Chen et al., 2014). Compared with ALFF, fractional ALFF (fALFF) shows much more sensitive and specific in detecting regional spontaneous neural activity during the resting state by suppressing the physiological noise (Zou et al., 2008). Therefore, fALFF may be a useful index to reflect the abnormal neural activity in specific brain regions of tinnitus patients. Regional homogeneity (ReHo) is a robust algorithm that quantifies the local synchronization within neighboring voxels during resting state (Zang et al., 2004), which can be used to detect abnormal local neural activity coherence across the whole brain. Altered ReHo values may reflect the disequilibrium of spontaneous neural activity within and between corresponding brain regions. Indeed, aberrant ReHo values, indicative of disrupted local functionality, have been linked to several neurological impairments (Liu et al., 2006; He et al., 2007; Wu et al., 2009; Chen et al., 2012b). Therefore, ReHo measurement is a potentially powerful tool to detect aberrant resting-state brain activity. Based on the main brain regions involved in tinnitus, the ROI-based FC analysis is appropriate, which could accurately explore the characteristics of FC changes in brain regions directly related to tinnitus, and reflects the characteristics of brain network alterations. In previous studies, the auditory network, visual network, attention network, control network and default mode network (DMN) have been reported involving in tinnitus using ROI-based FC (Burton et al., 2012; Husain and Schmidt, 2014; Leaver et al., 2016; Chen et al., 2017a, b, 2018a). These studies have confirmed that FC is an effective method for studying the synchronization of functional activities in different brain regions of tinnitus, which could better reflect the abnormal FC changes in tinnitus brain regions. These three techniques can directly measure the intensity and consistency of neural activity and FC changes between brain regions and have high test-retest reliability. Thus, the combination of these three methods may detect more comprehensively local and regional neural functional changes than either method alone.

As we described above, chronic tinnitus patients showed altered resting-state brain networks including auditory network, visual network, attention network, control network, and DMN. However, the alterations of brain network in AT patients were poorly understood. To achieve this goal, we combined fALFF, ReHo, and FC to explore brain functional abnormalities in AT patients with hearing loss. The study focused on two main hypotheses: (1) altered fALFF, ReHo and FC would be observed between tinnitus patients and a healthy control group; (2) the abnormal resting-state networks would be correlated with specific tinnitus characteristics.

## Materials and Methods

### Subjects

This study included AT and a healthy control (HC) group with 59 right-handed subjects with at least 8 years of education. Patients were recruited at the Affiliated Jiangning Hospital, Nanjing Medical University from February 2018 to March 2019, including inpatients and outpatients. The selected patients had constant, unilateral tinnitus with sensorineural hearing loss (SHL) in one ear, duration <1 month. The healthy adults were recruited through community health screenings or newspaper advertisements and were matched with tinnitus patients with respect to age, sex, and education. The AT group had 28 (14 male) patients. Among them, 12 patients have tinnitus in the right ear and 16 patients with left ear tinnitus. The HC group consisted of 31 (19 male) subjects. Pure-tone audiometry (PTA) was performed to measure the hearing level at the frequencies of 125, 250, 500, 1000, 2000, 4000, and 8000 Hz. All patients were included if the mean PTA hearing threshold at the frequencies from 0.125 to 8 kHz was greater than 30 dB in one ear. All healthy control group subjects had normal hearing (defined as hearing loss of no more than 25 dB at any of the 7 frequencies). Additionally, individuals were excluded from the study if they had any of these situations, namely, Meniere’s disease, pulsatile tinnitus, hyperacusis, a past history of severe alcoholism, smoking, head injury, stroke, Alzheimer’s disease, Parkinson’s disease, epilepsy, major depression or other neurological or psychiatric illness, major medical illness (e.g., cancer, anemia, and thyroid dysfunction), MRI contraindications or severe visual loss (Chen et al., 2018b). All the subjects provided written informed consent before their participation in the study protocol, which was approved by the Research Ethics Committee of the Nanjing Medical University.

All patients were required to accomplish the tinnitus handicap inventory (THI) questionnaire to assess the severity of their tinnitus (Newman et al., 1996). Higher total scores (from a range of 0 to 100) represent a higher impact of tinnitus on daily life. None of the participants had depression and anxiety according to the self-rating depression scale (SDS) and self-rating anxiety scale (SAS) (overall scores <50 for each). The detailed clinical characteristics and demographics of all participants were listed in Table 1.

**TABLE 1:** Demographic and clinical characteristics of participants.

	**ATs (*n* = 28)**	**HCs (*n* = 31)**	***P* value**
Age (years)	41.2 ± 11.61	45.4 ± 14.32	0.22
Genger (male/female)	14/14	12/19	0.40
Education (years)	13.6 ± 7.34	13.3 ± 3.31	0.81
Handedness (right/left)	28/0	32/0	1.00
Tinnitus laterality (right/left)	12/16	–	–
Duration (days)	7.79 ± 6.56	–	–
PTA of right ear (dB)	60.24 ± 13.56	14.2 ± 2.54	<0.001^∗^
PTA of left ear (dB)	58.52 ± 14.21	14.6 ± 2.62	<0.001^∗^
THI score	30.43 ± 11.89	–	–
SAS score	29.29 ± 4.67	27.32 ± 4.56	0.26
SDS score	27.71 ± 4.98	26.21 ± 4.63	0.33

*Data are presented as mean ± SD. PTA, pure-tone audiometry; THI, tinnitus handicap inventory; SAS, self-rating anxiety scale; SDS, self-rating depression scale. ^∗^*P* < 0.001 (independent-sample *t*-test, two-tailed), showed statistical difference in hearing threshold between ATs and HCs.*

### MRI Data Acquisition

All patients were scanned before any drug treatment. The imaging data were acquired on a 3.0 T MRI scanner (Ingenia, Philips Medical Systems, Netherlands) with an 8-channel receiver array head coil. Head motion and scanner noise were reduced using foam padding and earplugs. All participants were asked to rest quietly with their eyes closed but to remain awake and were instructed not to focus their thoughts on anything in particular during the MR acquisition. First, a three-dimensional turbo fast echo (3D-TFE) T1 WI sequence with high resolution was performed to acquire structural images as follows: repetition time (TR)/echo time (TE) = 8.1/3.7 ms; slices = 170; thickness = 1 mm; gap = 0 mm; flip angle (FA) = 8°; acquisition matrix = 256 × 256; and field of view (FOV) = 256 mm × 256 mm. The structural sequence took 5 min and 29 s. Based on the anatomical images, functional images were obtained with a gradient echo-planar imaging sequence as follows: TR = 2000 ms; TE = 30 ms; slices = 36; thickness = 4 mm; gap = 0 mm; FOV = 240 mm × 240 mm; acquisition matrix = 64 × 64; and FA = 90°. The fMRI sequence took 8 min and 8 s. Finally, the conventional axial T2WI (TR = 4000 ms; TE = 107 ms; slices = 18; thickness = 5 mm; gap = 1.5 mm; FOV = 230 mm × 230 mm; acquisition matrix = 384 × 384; and FA = 90°) and sagittal T2WI FLAIR (TR = 10000 ms; TE = 120 ms; slices = 18; thickness = 5 mm; gap = 1.5 mm; FOV = 220 mm × 220 mm; acquisition matrix = 336 × 189) sequences are performed for excluding intracranial organic lesions.

### MRI Data Analyses

#### Data Preprocessing

Data analyses were performed using the toolbox for Data Processing & Analysis for Brain Imaging (DPABI V3.1^1^) (Yan et al., 2016), which is based on Statistical Parametric Mapping (SPM8^2^). After removing the first 10 volumes, slice-timing, realignment for head motion correction, and nuisance covariate regression were performed. Afterward, the images were spatially normalized to the Montreal Neurological Institute template (resampling voxel size = 3 mm^3^ × 3 mm^3^ × 3 mm^3^). Any subjects with a head motion of >2.0 mm translation or 2.0° rotation in any direction were excluded.

#### fALFF Analysis

After smoothing (FWHM = 4 mm), detrending and filtering (0.01–0.08 Hz), time courses were converted to the frequency domain using fast Fourier transform. Then, ALFF was computed by taking the square root of the power spectrum, then averaging and squaring across 0.01–0.08 Hz at each voxel. At last, fALFF was calculated as the ALFF divided by the value of the entire detectable frequency band. Two-sample *t*-tests were performed by using DPABI software (within a group mask) to calculate the fALFF difference between groups, with age, sex, education level, and gray matter (GM) volume as covariates. The result was determined by AlphaSim correction (*P* < 0.001 at the voxel level and *P* < 0.01 at the cluster level). Previous rs-fMRI study provided considerable evidences for abnormal intrinsic brain activity between patients with left-sided hearing impairment and those with right-sided hearing impairment (Xie et al., 2019), which makes this seem like a worthwhile question to explore. Thus, we presented unthresholded t-stat fALFF image for left and right-sided groups (uncorrected, *P* < 0.05, cluster size >20 voxels; Age, sex, education level, and GM volume were included as nuisance covariates).

#### ReHo Analysis

After bandpass filtering (0.01–0.08 Hz), Kendall’s coefficient of concordance was performed to calculate the local synchronization of a given voxel with its 26 nearest neighbors (Zang et al., 2004). Then we standardized the ReHo value of each voxel by partitioning the primal value using the global mean ReHo value. After that, the normalized data were smoothed with a 4 mm full-width at half maximum (FWHM) Gaussian kernel for further statistical analysis. To explore between-group differences, a two-sample *t*-tests were performed under DPABI software. Age, sex, education level, and GM volume were included as nuisance covariates, to control for the possible influences of these factors on the results. A threshold was used by AlphaSim correction (*P* < 0.001 at the voxel level and *P* < 0.001 at the cluster level). As we described in fALFF analysis, the unthresholded t-stat ReHo image was also performed for left and right-sided groups (uncorrected, *P* < 0.05, cluster size >20 voxels; Age, sex, education level, and GM volume were included as nuisance covariates).

#### FC Analyses

Resting-state FC was also conducted under DPABI software. Based on the fALFF and ReHo findings in the brain regions between groups, we defined six ROIs including the right ITG, the vermis_8, the right calcarine cortex, the right precuneus, the right supramarginal gyrus (SMG), and the right middle frontal gyrus (MFG). The mean time series of each ROI was obtained for the reference time course. Then, Pearson’s correlation coefficients were calculated between the mean signal change of each ROI and the time series of each voxel. Finally, a Fisher’s *z*-transform was applied to improve the normality of the correlation coefficients (Lowe et al., 1998). Six head motion parameters and mean time series of global, white matter, and cerebrospinal fluid signals were included in the regression analysis to eliminate possible effects of such factors on the results. Two-sample *t*-tests were performed to explore the differences of the FC of each ROI between tinnitus patients and controls. Age, sex, education level, and GM volume were included as covariates. AlphaSim correction was used for the multiple comparisons (*P* < 0.001 at the voxel level and *P* < 0.01 at the cluster level).

### Statistical Analysis

Independent two-sample *t* tests and Chi-square tests were performed using SPSS 22.0 software to compare general data and scale scores (*P* < 0.05 were considered to be significant). To explore the abnormalities of the fALFF, ReHo and FC maps between AT and HC group, two-sample *t* tests were analyzed by using DPABI software (within a Group mask). Additionally, we regressed out the impact of age, sex, education level, and GM volume. AlphaSim was used for the multiple comparisons (ReHo analysis: *P* < 0.001 at the voxel level and *P* < 0.001 at the cluster level; fALFF and FC analysis: *P* < 0.001 at the voxel level and *P* < 0.01 at the cluster level). The differences between left and right-sided group were conducted under two-sample *t* tests in DPABI software (within a Group mask), which was uncorrected (*P* < 0.05, cluster size >20 voxels). In addition, age, sex, education level, and GM volume were included as covariates. Pearson correlation analyses and Partial correlation analyses were then conducted between altered fALFF values, ReHo values, FC z-values and AT characteristics in the AT group using SPSS software. The Bonferroni correction for multiple comparisons was applied (*P* < 0.05 were considered statistically significant).

## Results

### fMRI Data Analyses

**FIGURE 1:**
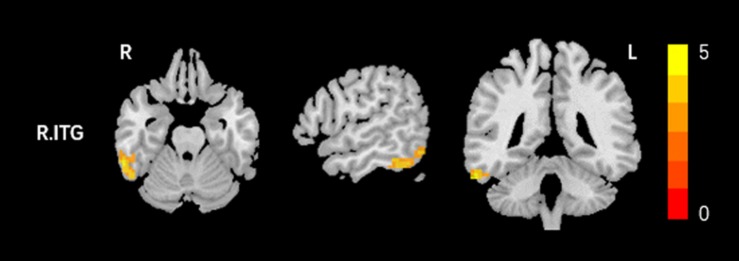
Increased fALFF values between tinnitus patients and healthy controls (HCs). The threshold was determined by AlphaSim correction (*P* < 0.001 at the voxel and *P* < 0.01 at the cluster level). R, right; ITG, inferior temporal gyrus. Red to yellow means Increased fALFF values.

**FIGURE 2:**
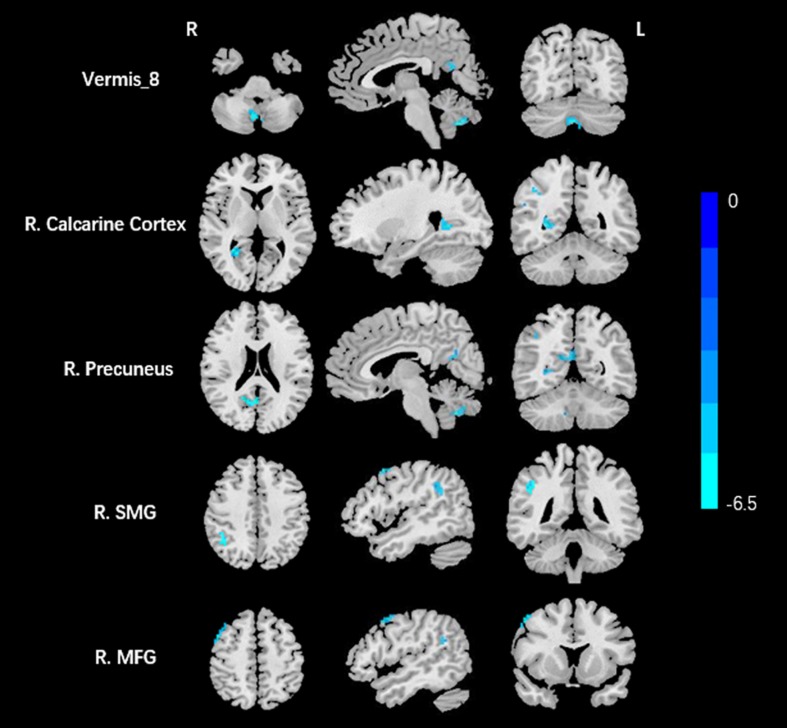
Decreased ReHo values between tinnitus patients and HCs. The threshold was determined by AlphaSim correction (*P* < 0.001 at the voxel and *P* < 0.01 at the cluster level). R, right; SMG, supramarginal gyrus; MFG, middle frontal gyrus. Blue means decreased ReHo values.

Compared to the HCs, AT had significantly increased fALFF values in the right ITG (Figure 1 and Table 2). We did not find any decreased regions between ATs and HCs in the fALFF map. At the same time, AT patients had lower ReHo values in the cerebellar vermis, the right calcarine cortex, the right precuneus, the right SMG, and the right MFG (Figure 2 and Table 2). Moreover, no increased ReHo values were observed in the study. We also did not find any abnormal fALFF and ReHo values in auditory regions.

**TABLE 2:** fALFF and ReHo differences between tinnitus and healthy controls.

	**Brain region**	**Hemisphere**	**Peak MNI coordinates**	**T score**	**Cluster size (voxels)**
			**(x,y,z)**		
fALFF	Inferior temporal gyrus	*R*	60, −42, −24	4.8772	77
ReHo	Vermis_8	–	3, −69, −39	−6.089	27
	Calcarine cortex	R	27, −51, 6	−6.5194	22
	Precuneus	R	0, −57, 21	−5.2884	30
	Supramarginal gyrus	R	42, −42, 42	−5.2124	40
	Middle frontal gyrus	R	48, 12, 54	−5.7163	31

*fALFF analysis: The threshold was determined by AlphaSim correction (*P* < 0.001 at the voxel and *P* < 0.01 at the cluster level); ReHo analysis: The threshold was determined by AlphaSim correction (*P* < 0.001 at the voxel and *P* < 0.01 at the cluster level). L, left; R, right; MNI, montreal neurological institute.*

Compared with the right-sided acute tinnitus group, the left-sided group showed significantly higher fALFF values in the right cerebellar, bilateral middle temporal gyrus (MTG), left ITG, left triangular part inferior frontal gyrus, left superior frontal gyrus (SFG), and left precentral gyrus; moreover, the left-sided acute tinnitus group showed significantly lower fALFF values in the left postcentral gyrus (Supplementary Figure S1 and Supplementary Table S1). Compared with the right-sided acute tinnitus group, the left-sided group showed significantly higher ReHo values in the bilateral cerebellar, left fusiform gyrus, left MTG, left MFG, left medial superior frontal gyrus, right SMG, and right SFG; moreover, the left-sided acute tinnitus group showed significantly lower ReHo values in the left postcentral gyrus (Supplementary Figure S2 and Supplementary Table S1). Unfortunately, there were no clusters survived under multiple comparison correction.

**FIGURE 3:**
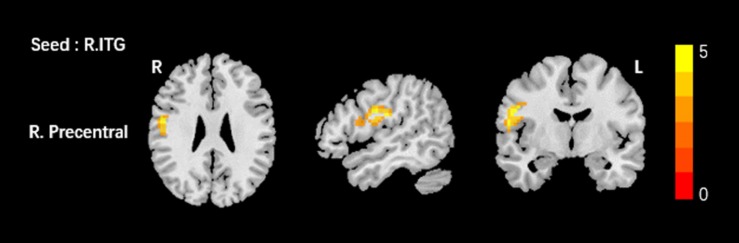
Comparing to HCs, patients showed enhanced connectivity between the seed region in the right ITG and the right precentral gyrus. The threshold was determined by AlphaSim correction (*P* < 0.001 at the voxel and *P* < 0.01 at the cluster level). R, right; ITG, inferior temporal gyrus. Red to yellow means increased connectivity.

**FIGURE 4:**
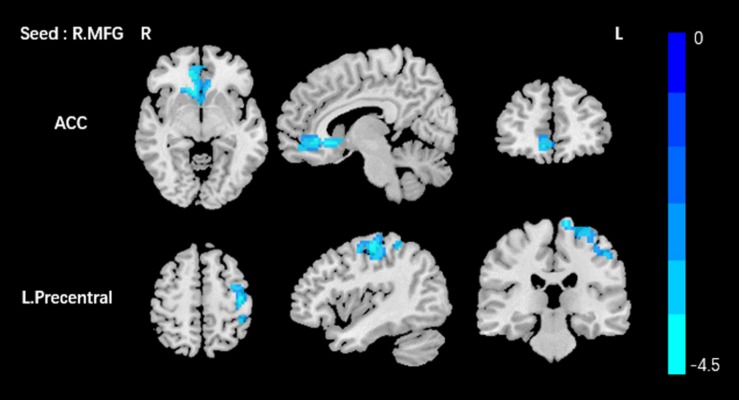
Comparing to HCs, patients showed reduced connectivity between the seed region in the right MFG, and the bilateral ACC, left precentral gyrus. The threshold was determined by AlphaSim correction (*P* < 0.001 at the voxel and *P* < 0.01 at the cluster level). R, right; L, left; MFG, middle frontal gyrus; ACC anterior cingulate cortex. Blue means decreased connectivity.

Based on the increased fALFF and decreased ReHo values in the tinnitus patients, six ROIs were defined for our connectivity analysis: the right ITG of the fALFF map, and the vermis_8, the right calcarine cortex, left precuneus, right SMG, and right MFG of the ReHo map. When we set the seed region in the vermis_8, the right calcarine cortex, the left precuneus, and the right SMG, and there were no significantly altered connectivity found after multiple comparisons correction by using AlphaSim. Compared to the control group, tinnitus patients showed significantly enhanced connectivity between the seed region in the right ITG and the right precentral gyrus (Table 3 and Figure 3). Additionally, the seed region in the right MFG exhibited decreased connectivity with the bilateral anterior cingulate cortex (ACC) and the left precentral gyrus in AT patients (Table 3 and Figure 4).

**TABLE 3:** Regions showing seed-based FC differences between tinnitus patients and normal controls.

**Seed region**	**Brain region**	**Hemisphere**	**Peak MNI coordinates**	**T score**	**Cluster size (voxels)**
			**(x,y,z)**		
R.MFG	Anterior cingulate cortex	B	9, 21, −6	−4.2742	118
	Precentral gyrus	L	−12, −27, 75	−4.5977	148
R.ITG	Precentral gyrus	R	51, −6, 7	4.7588	62

*The threshold was determined by AlphaSim correction (*P* < 0.001 at the voxel and *P* < 0.01 at the cluster level). MNI, montreal neurological institute; L, left; R, right; B, bilateral; MFG, middle frontal gyrus; ITG, inferior temporal gyrus.*

### Correlation Analyses

Surprisingly, in tinnitus patients, we did not find any significant correlations between fALFF values, ReHo values, FC, and tinnitus characteristics (including duration, THI, SAS, and SDS) neither controlled for age, sex, education, GM volume, average hearing thresholds nor the non-adjusted correlation analyses (Table 4 and Supplementary Table S2). Even so, we found that the increased fALFF values in right ITG was almost negatively correlated with SDS scores (*r* = −0.392, *p* = 0.052, Table 4).

**TABLE 4:** Correlation coefficients between altered fALFF, ReHo, FC values, and different tinnitus characteristics.

	**Brain region**	**Duration**	**THI**	**SDS**	**SAS**
fALFF	R.ITG	0.074/0.726	−0.175/0.404	−0.392/0.052	−0.056/0.792
ReHo	Vermis_8	−0.239/0.250	0.056/0.789	−0.121/0.566	−0.114/0.587
	R.Calcarine cortex	−0.142/0.499	0.177/0.397	0.003/0.989	−0.336/0.101
	R.Precuneus	−0.086/0.681	−0.152/0.468	−0.168/0.423	−0.257/0.216
	R.SMG	0.354/0.082	0.226/0.278	0.191/0.361	−0.142/0.497
	R.MFG	0.087/0.681	−0.168/0.422	−0.309/0.133	−0.206/0.322
FC	R.MFG-B.ACC	0.262/0.206	−0.018/0.933	0.196/0.348	−0.102/0.628
	R.MFG-L.Precentral gyrus	−0.004/0.984	0.352/0.084	0.137/0.512	0.091/0.664
	R.ITG-R.Precentral gyrus	−0.004/0.984	0.095/0.652	−0.271/0.190	−0.365/0.073

*Partial correlations were controlled for age, sex, education level, GM volume, and mean hearing thresholds (data are represented as r/p). *P* < 0.05 was considered significant. L, left; R, right; B, bilateral; MFG, middle frontal gyrus; ITG, inferior temporal gyrus; ACC, anterior cingulate cortex.*

## Discussion

The present study focused on the early stage of tinnitus. The brain function abnormalities in patients with unilateral acute tinnitus and hearing loss were detected through fALFF, ReHo, and FC analysis. The results demonstrated abnormal spontaneous brain activity in certain brain regions, for instance, the right ITG, the vermis, the right calcarine cortex, the bilateral precuneus, the right SMG and the right MFG. Additionally, based on the different brain regions in fALFF and ReHo, FC analysis showed enhanced connectivity between the right ITG (seed region) and the right precentral gyri, along with reduced connectivity between the right MFG (seed region) and the bilateral ACC, left precentral gyrus. Thus, our work clearly exhibited intra- and interregional changes in the neural function of patients with unilateral acute tinnitus and hearing loss.

The middle frontal gyrus, an important part of the prefrontal cortex, is believed to principally participates in the neural mechanisms of inhibiting long-term disturbance (Konishi et al., 2005). A ^18^F-fluoro-deoxyglucose positron emission tomography (FDG-PET) study indicated that tinnitus patients had enhanced FDG metabolism in the left MFG (Geven et al., 2014). Golm et al. (2013) demonstrated that the high neural activation in the left MFG was associated with tinnitus severity in chronic tinnitus patients based on a fMRI study. Structurally, Aldhafeeri et al. (2012) also found altered cortical thickness in the bilateral MFGs in patients with severe tinnitus. Therefore, it is believed that the neural activity in MFG in tinnitus may reflect the severity of the condition. Contrary to a previous study, our research found reduced ReHo values in the right MFG of AT. We speculated that it is probably related to the short duration of the tinnitus, as most of the tinnitus participants presented lower scores in the THI test. On the other hand, the various neuroimaging methods employed to study tinnitus (PET vs. fMRI) and the tinnitus patients’ heterogeneity (with or without hearing loss) may lead to inconsistent results. Based on the FC analysis, we detected that tinnitus patients had decreased connectivity between the seed region in the right MFG and the bilateral ACC. The MFG and ACC are primary parts of the DMN. The DMN is usually active when people are in the resting state and is suppressed when the subject enters a task-based state (Raichle et al., 2001). We detected that tinnitus patients had decreased connectivity between the seed region in the right MFG and the bilateral ACC. The DMN is involved in episodic memory, self-evaluation, social functioning, foresight, and intrinsic expression (Andrews-Hanna, 2012). Chen et al. (2015b) and Zhang et al. (2015) found tinnitus patients had altered spontaneous neural activity or disrupted functional connection within the DMN which was associated with the severity of the tinnitus. Therefore, the auditory hallucinations discovered in tinnitus may be correlated with abnormalities of the DMN, and the abnormal or incoherent brain activity in this study may reflect this abnormal network. Additionally, we noticed that the right MFG had decreased connectivity with the left precentral gyrus, which was reported to be associated with the executive control network in tinnitus (Chen et al., 2017b).

The enhanced activity of the ITG observed in our study suggests that non-auditory pathways involved in tinnitus might reflect abnormal sensory integration and memory consolidation processes. Indeed, the ITG is involved in memory consolidation, visual processing and multimodal sensory integration (Mesulam, 1998). Many studies in EEG and PET have shown that tinnitus patients have hyperexcitability of the bilateral ITG compared to normal controls (Weiler et al., 2000; Ashton et al., 2007; Pawlak-Osinska et al., 2013; Adamchic et al., 2014; Song et al., 2015). This abnormal activation, confirmed by our results, might be related to a mechanism of maladaptive memory consolidation and/or abnormal sensory integration (Song et al., 2012). Additionally, the right ITG showed enhanced connectivity with the right precentral gyrus.

Traditionally, the cerebellum was considered to be restricted to motor control and coordination, but previous studies have indicated that it also showed an important role in the detection of auditory afferents and processing (Parsons et al., 2009; Salmi et al., 2009). Our present study found that AT patients had decreased ReHo values in the cerebellar vermis, and it is reported that the cerebellum is involved in receiving and processing information input from auditory-related cortices (Petacchi et al., 2005). We thus presume that the cerebellum may be related to the processing of tinnitus-related auditory hallucinations. Furthermore, an fMRI study found disrupted connectivity between the auditory cortex and the cerebellum (Maudoux et al., 2012a), which was in consistent with a salicylate tinnitus rat model study (Chen et al., 2015a). According to previous studies, we presumed that the cerebellum may participate in contrasting and integrating afferent signals from the cochlea and output signals from the auditory cortex in tinnitus patients, that is, acting as a selective gating control (Bauer et al., 2013), but further studies are needed for verification.

The calcarine cortex is also known as an important part of the primary visual cortex, which is the main site of input of signals coming from the retina. Our study indicated that AT showed decreased neural activity consistently in the right calcarine cortex. Chen et al. (2014) found decreased ALFF values in the visual area in chronic tinnitus patients. Anatomically, animal studies demonstrated an connection between auditory and visual regions (Iurilli et al., 2012), and the auditory cortex directly regulated the visual cortex (Ibrahim et al., 2016). Maudoux et al. (2012b) found functional connections between auditory and visual subnetworks in tinnitus patients. One interpretation of these results is that as patients attend to their phantom auditory sensation, they contemporaneously activate visual areas.

The supramarginal gyrus, a region associated with the attention network (Rushworth et al., 1997; Yamasaki et al., 2002), was found to have reduced ReHo value in AT patients. Previous EEG or PET studies also indicated that the SMG may play a role in tinnitus (Mirz et al., 1999; Weiler et al., 2000). Based on seed-FC analysis, Schmidt et al. (2013) demonstrated that the dorsal attention network (DAN) showed a declined relationship with the right SMG in tinnitus subjects. Hence, the decreased local neural activity in the SMG may change the connectivity in the DAN in tinnitus patients. This evidence indicates that the altered intraregional brain activity in the attention network might lead to abnormal local synchronization in tinnitus.

In the present study, we also found lower spontaneous brain activity in the precuneus. Prior studies showed that the precuneus is related to conscious and internal awareness (Cavanna and Trimble, 2006; Cavanna, 2007; Vanhaudenhuyse et al., 2011) and thus may play a role in tinnitus generation and persistence. Several hypotheses have suggested that conscious awareness is necessary for tinnitus perception. However, the percept can be effectively ignored if distractors are provided (Roberts et al., 2013). Tinnitus habituation may therefore be linked to networks of awareness, of which the precuneus plays a key role. Additionally, the precuneus is also an important structure of the DMN and it suggested that intrinsic alteration of DMN was involved in acute tinnitus.

Interestingly, almost all the alterations in ReHo, fALFF, and FC were lateralized to the right hemisphere in patients. A number of functional and structural studies in tinnitus demonstrated a lateralized effect (Smits et al., 2007; Schecklmann et al., 2013; Chen et al., 2014, 2016, 2018b; Geven et al., 2014), some of them biased to the left and others to the right. We speculate that the cause of lateralization may due to the heterogeneity of tinnitus patients or the different neuroimaging methods used to investigate tinnitus. Therefore, the observed right-lateralization effect needs to be studied to determine whether it is related specifically to tinnitus or other factors.

In the correlation analyses, we failed to observe significant differences between fALFF values, ReHo values, FC and tinnitus characteristics. But we found that the increased fALFF values in right ITG was almost negatively correlated with SDS scores. We speculate that this might be associated with the short duration of the tinnitus in the patients. Furthermore, the method of neuroimaging we used may be insensitive to the early stages of tinnitus.

The present study has several constraints that must be acknowledged. First, because of the small sample size of tinnitus patients, we could not categorize the group into right/left side tinnitus to verify whether the bias of the tinnitus lead to the lateralization of functional activity. Although we performed the differences between left and right-side tinnitus in a exploratory way, it still needs specific work to verify. Second, due to the cross-sectional design, we could not study the changes on the brain impacted by disease and whether the neural tinnitus network will change over time. Therefore, a longitudinal study involving a larger number of participants will be needed for future research. Third, although we used earplugs to minimize noise effects in present study, the noise of the scanner could not be completely prevented. Thus, this limitation may reduce the differences in resting-state networks between tinnitus and control groups. Furthermore, although the seed-based FC method is a powerful tool to study the integration of distributed brain systems, it still raises concerns of double-dipping. Finally, in addition to functional alterations, more studies are needed to detect the potentially structural connectivity in acute tinnitus, in our knowledge, diffusion tensor imaging (DTI) may measure that more visually.

## Conclusion

By combining ReHo, fALFF, and FC analyses, our work indicated that AT with hearing loss had abnormal intraregional neural activity and disrupted connectivity in several brain regions which mainly including the non-auditory area, and these regions are major components of DMN, attention network, visual network, and executive control network. These findings will help us enhance the understanding of the neuroimaging mechanism in tinnitus populations. Moreover, these abnormalities remind us that we should focus on the early stages of this hearing disease.

## Data Availability

The datasets generated for this study are available on request to the corresponding author.

## Ethics Statement

This study was approved by the Ethics Committee of Nanjing Medical University. All subjects were informed about the purpose of the study before giving their written consent.

## Author Contributions

G-PZ and X-YS collected the fMRI data, performed the analysis, and wrote the manuscript. Y-SY helped with data collection. H-LW and L-JQ contributed to the fMRI data analysis and discussion. Q-QZ helped with data processing. XY revised this manuscript. HZ and Y-JT designed the MRI experiment and manuscript revision.

## Conflict of Interest Statement

The authors declare that the research was conducted in the absence of any commercial or financial relationships that could be construed as a potential conflict of interest.

## Footnotes

1 http://rfmri.org/dpabi

2 http://www.fil.ion.ucl.ac.uk/spm

## Supplementary Material

FIGURE S1 Brain regions showed fALFF differences between left and right-sided acute tinnitus group (uncorrected, *P* < 0.05, cluster size >20 voxels). The regions with higher fALFF values were right cerebellar, bilateral MTG, left ITG, left triangular part inferior frontal gyrus, left SFG,and left precentral gyrus. The regions with lower fALFF values were left postcentral gyrus. MTG, middle temporal gyrus; ITG, inferior temporal gyrus; SFG, superior frontal gyrus. Yellow color means increased fALFF values and blue color means decreased fALFF values.

FIGURE S2 Brain regions showed ReHo differences between left and right-sided acute tinnitus group (uncorrected, *P* < 0.05, cluster size >20 voxels). The regions with higher ReHo values were bilateral cerebellar, left fusiform gyrus, left MTG, left MFG, left medial superior frontal gyrus, right SMG, and right SFG. The regions with lower ReHo values were left postcentral gyrus. MTG, middle temporal gyrus; MFG, middle frontal gyrus; SMG, supramarginal gyrus. Yellow color means increased ReHo values and blue color means decreased ReHo values.

TABLE S1 Brain regions showing fALFF and ReHo differences between left and right-sided tinnitus fALFF and ReHo analysis: *P* < 0.05 (uncorrected), cluster size >20 voxels; Age, sex, education level, and grey matter (GM) volume were included as nuisance covariates. L, left; R, right; B, bilateral. MNI, montreal neurological institute.

TABLE S2 Pearson correlation analyses between altered fALFF, ReHo, FC values and different tinnitus characteristics Pearson correlation analyses were conducted without controlling for age, sex, education level, GM volume and mean hearing thresholds (Data are represented as r/p). *P* < 0.05 was considered significant. L, left; R, right; B, bilateral; MFG, middle frontal gyrus; ITG, inferior temporal gyrus; ACC, anterior cingulate cortex.
