# Supplementary figures and images for: Disrupted Intraregional Brain Activity and Functional Connectivity in Unilateral Acute Tinnitus Patients With Hearing Loss
Source: Front Neurosci. 2019 Sep 19;13:1010. doi: 10.3389/fnins.2019.01010 (PMC6761222; doi:10.3389/fnins.2019.01010)

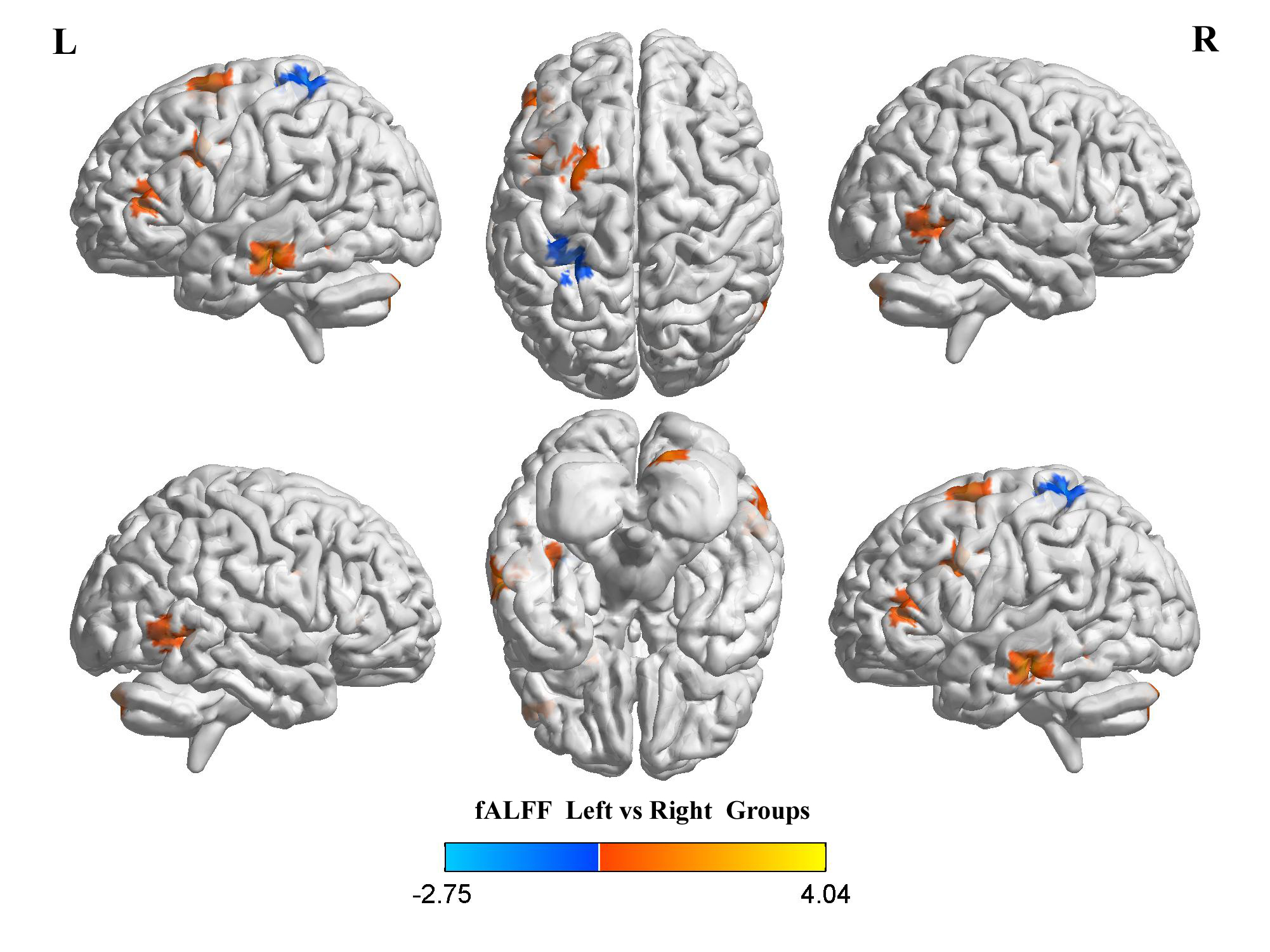

Supplement: FIGURE S1 — Brain regions showed fALFF differences between left and right-sided acute tinnitus group (uncorrected, P < 0.05, cluster size >20 voxels). The regions with higher fALFF values were right cerebellar, bilateral MTG, left ITG, left triangular part inferior frontal gyrus, left SFG,and left precentral gyrus. The regions with lower fALFF values were left postcentral gyrus. MTG, middle temporal gyrus; ITG, inferior temporal gyrus; SFG, superior frontal gyrus. Yellow color means increased fALFF values and blue color means decreased fALFF values. [file Image_1.JPEG]

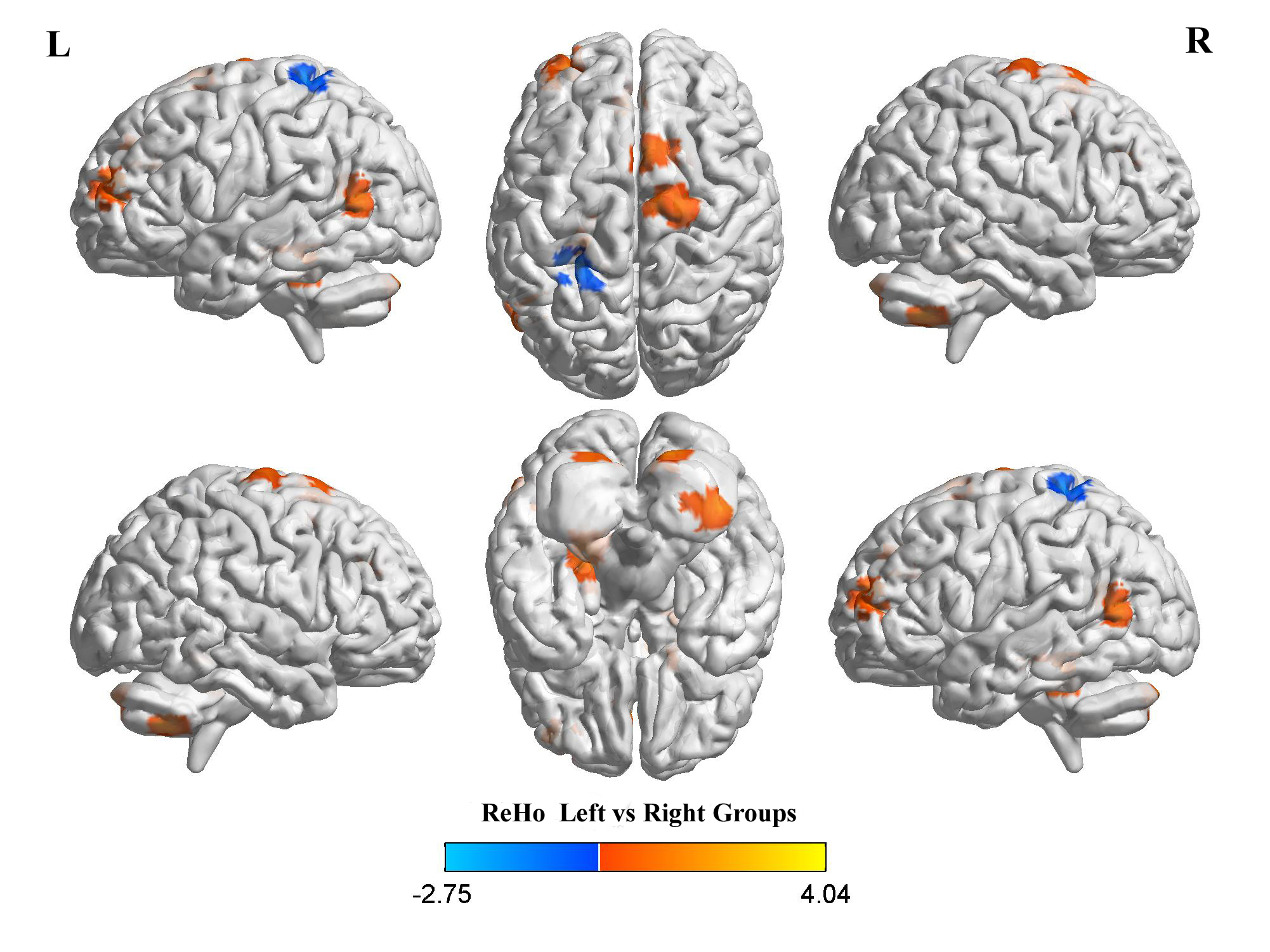

Supplement: FIGURE S2 — Brain regions showed ReHo differences between left and right-sided acute tinnitus group (uncorrected, P < 0.05, cluster size >20 voxels). The regions with higher ReHo values were bilateral cerebellar, left fusiform gyrus, left MTG, left MFG, left medial superior frontal gyrus, right SMG, and right SFG. The regions with lower ReHo values were left postcentral gyrus. MTG, middle temporal gyrus; MFG, middle frontal gyrus; SMG, supramarginal gyrus. Yellow color means increased ReHo values and blue color means decreased ReHo values. [file Image_2.JPEG]
