# Supplementary material for: Disrupted Intraregional Brain Activity and Functional Connectivity in Unilateral Acute Tinnitus Patients With Hearing Loss
Source: Front Neurosci. 2019 Sep 19;13:1010. doi: 10.3389/fnins.2019.01010 (PMC6761222; doi:10.3389/fnins.2019.01010)
Supplement: TABLE S1 — Brain regions showing fALFF and ReHo differences between left and right-sided tinnitus fALFF and ReHo analysis: P < 0.05 (uncorrected), cluster size >20 voxels; Age, sex, education level, and grey matter (GM) volume were included as nuisance covariates. L, left; R, right; B, bilateral. MNI, montreal neurological institute. [file Table_1.DOCX]

**Table S1 Brain regions showing fALFF and ReHo differences between left and right-sided tinnitus**

|  | Brain region | Hemisphere | Peak MNI coordinates | T score | Cluster Size  (voxels) |
| --- | --- | --- | --- | --- | --- |
|  |  |  | (x,y,z) |  |  |
| ReHo | Cerebelum_Crus2_R | - | 39, -60, -48 | 4.0851 | 32 |
|  | Cerebelum_Crus2_L | - | -6, -84, -33 | 3.4849 | 37 |
|  | Fusiform Gyrus | L | -21, -30, -18 | 3.5021 | 42 |
|  | Middle Temporal Gyrus | L | -45, -54, 18 | 3.5328 | 27 |
|  | Middle Frontal Gyrus | L | -36, 51, 12 | 3.3879 | 31 |
|  | Medial Superior Frontal Gyrus | L | 0, 42, 33 | 3.1218 | 21 |
|  | Supplementary Motor Area | R | 12, 15, 66 | 2.969 | 27 |
|  | Postcentral Gyrus | L | -27, -42, 69 | -4.4391 | 24 |
|  | Superior Frontal Gyrus | R | 18, -9, 72 | 3.342 | 24 |
| fALFF | Cerebelum_Crus2_R | - | 15, -84, -36 | 3.1765 | 29 |
|  | Middle Temporal Gyrus | B | -66, -24, -18 | 3.7469 | 39 |
|  |  |  | 57, -60, 0 | 2.6625 | 25 |
|  | Inferior Temporal Gyrus | L | -42, -36, -15 | 3.0141 | 20 |
|  | Triangular part Inferior Frontal Gyrus | L | -45, 33, 9 | 3.0394 | 21 |
|  | Precentral Gyrus | L | -45, 6, 39 | 3.4732 | 42 |
|  | Superior Frontal Gyrus | L | -24, 6, 66 | 4.0356 | 22 |
|  | Postcentral Gyrus | L | -27, -42, 66 | -2.7539 | 22 |

fALFF and ReHo analysis: P < 0.05 (uncorrected), cluster size > 20 voxels; Age, sex, education level, and grey matter (GM) volume were included as nuisance covariates. L, left; R, right; B, bilateral. MNI, Montreal Neurological Institute.
