# Supplementary material for: Disrupted Intraregional Brain Activity and Functional Connectivity in Unilateral Acute Tinnitus Patients With Hearing Loss
Source: Front Neurosci. 2019 Sep 19;13:1010. doi: 10.3389/fnins.2019.01010 (PMC6761222; doi:10.3389/fnins.2019.01010)
Supplement: TABLE S2 — Pearson correlation analyses between altered fALFF, ReHo, FC values and different tinnitus characteristics Pearson correlation analyses were conducted without controlling for age, sex, education level, GM volume and mean hearing thresholds (Data are represented as r/p). P < 0.05 was considered significant. L, left; R, right; B, bilateral; MFG, middle frontal gyrus; ITG, inferior temporal gyrus; ACC, anterior cingulate cortex. [file Table_2.DOCX]

**Table S2 Pearson correlation analyses between altered fALFF, ReHo, FC values and different tinnitus characteristics**

|  | Brain region | Duration | THI | SDS | SAS |
| --- | --- | --- | --- | --- | --- |
| fALFF | R.ITG | -0.064/0.747 | 0.066/0.737 | 0.093/0.637 | 0.294/0.129 |
| ReHo | Vermis_8 | -0.003/0.988 | -0.134/0.496 | -0.302/0.114 | -0.319/0.093 |
|  | R.Calcarine Cortex | -0.169/0.391 | 0.213/0.275 | 0.025/0.899 | -0.226/0.248 |
|  | R.Precuneus | -0.209/0.285 | 0.088/0.657 | -0.101/0.608 | -0.117/0.553 |
|  | R.SMG | 0.336/0.080 | 0.203/0.301 | 0.082/0.677 | -0.154/0.435 |
|  | R.MFG | 0.018/0.926 | -0.019/0.924 | -0.047/0.812 | 0.024/0.902 |
| FC | R.MFG-B.ACC | 0.312/0.103 | -0.134/0.496 | -0.082/0.679 | -0.272/0.162 |
|  | R.MFG-L.Precentral Gyrus | 0.128/0.516 | 0.189/0.336 | -0.072/0.715 | -0.094/0.636 |
|  | R.ITG-R.Precentral Gyrus | 0.048/0.808 | 0.079/0.691 | -0.269/0.167 | -0.341/0.076 |

Pearson correlation analyses were conducted without controlling for age, sex, education level, GM volume and mean hearing thresholds (Data are represented as r/p). P < 0.05 was considered significant. L, left; R, right; B, bilateral; MFG, Middle Frontal Gyrus; ITG, Inferior Temporal Gyrus; ACC, Anterior Cingulate Cortex.
